# Safety and efficacy of the combination of selpercatinib with osimertinib in NSCLC: a case report and review of the literature

**DOI:** 10.3389/fonc.2025.1580322

**Published:** 2025-05-09

**Authors:** Blerina Resuli, Heidi Galarza, Diego Kauffmann-Guerrero, Julian Oversohl, Jürgen Behr, Amanda Tufman

**Affiliations:** ^1^ Department of Medicine V, University Hospital, Ludwig-Maximilans-University (LMU) Munich, Munich, Germany; ^2^ Comprehensive Pneumology Center Munich (CPC-M), German Center for Lung Research (DZL), Munich, Germany

**Keywords:** non-small cell lung cancer, EGFR, osimertinib, selpercatinib, EGFR-TKI acquired resistance

## Abstract

Osimertinib, a third-generation epidermal growth factor receptor–tyrosine kinase inhibitor (EGFR-TKI), has demonstrated significant clinical activity and tolerability in EGFR-positive non-small cell lung cancer (NSCLC) patients. However, acquired resistance to osimertinib is inevitable, and the mechanisms underlying resistance to third-generation EGFR-TKIs remain complex and not fully understood. In this case, we report a patient with EGFR-mutated NSCLC who progressed on first-line osimertinib treatment, acquiring a nuclear receptor coactivator 4–rearranged during transfection (NCOA4–RET) fusion alongside a co-mutation in the tumor protein p53 (TP53) gene. Despite these genomic alterations, the patient derived notable clinical benefit from the combination of osimertinib and selpercatinib, suggesting that this dual therapy may offer a promising approach to overcoming resistance in such cases.

## 1 Introduction

Activating mutations in the *epidermal growth factor receptor* (EGFR) gene are identified in approximately 15%–20% of patients with metastatic lung adenocarcinoma in Europe and the United States, with the most prevalent alterations being exon 19 deletions (most commonly E746_A750del) and the L858R point mutation in exon 21 (1). Based on the results of the FLAURA trial, the third-generation EGFR–tyrosine kinase inhibitor (EGFR-TKI) osimertinib has become the global standard of care for the first-line treatment of advanced EGFR-mutant non-small cell lung cancer (NSCLC), demonstrating significant improvements in both progression-free and overall survival compared to earlier-generation TKIs (2). Despite its efficacy, acquired resistance to osimertinib inevitably develops. Known mechanisms of resistance include on-target secondary EGFR mutations (such as C797S), as well as off-target bypass signaling alterations, most notably mesenchymal–epithelial transition (MET) factor amplification and *rearranged during transfection* (RET) gene fusions (3). RET fusions, although relatively rare, have emerged as a clinically relevant resistance mechanism in the post-osimertinib setting. These fusions result in constitutive activation of the RET tyrosine kinase domain through linkage with upstream partner genes that often contain strong promoter and dimerization domains, enhancing RET signaling independent of its native ligand. The most frequently reported fusion partners in NSCLC include *kinesin family member 5B–RET* (KIF5B–RET), which is the most prevalent RET fusion in *de novo* RET-rearranged NSCLC, accounting for approximately 70%–80% of RET fusion-positive cases in the treatment-naïve setting; *coiled-coil domain containing 6–RET* (CCDC6–RET), which accounts for 10%–20% of RET fusions and is more commonly observed in acquired resistance contexts following targeted therapy; and *nuclear receptor coactivator 4–RET* (NCOA4–RET), which is a less frequent fusion partner, representing approximately 5%–10% of cases.

A multicenter study by Rotow et al. (4) characterized RET fusions as an acquired resistance mechanism in a cohort of 14 patients who had progressed on osimertinib. Importantly, the combination of osimertinib with selpercatinib, a selective and potent RET inhibitor, demonstrated encouraging clinical activity and an acceptable safety profile in this context, supporting a rationale for the dual inhibition of both the EGFR and RET pathways.

The prognostic implications of RET fusions as a resistance mechanism remain an area of ongoing investigation. While RET fusions confer a clear mechanism of resistance to osimertinib, early evidence suggests that patients harboring RET fusions may experience longer post-progression survival compared to those with EGFR C797S or histologic transformation, possibly due to the availability of effective targeted therapies, such as selpercatinib and pralsetinib, which show high response rates in RET fusion-positive tumors. In the previously cited study, the combination of osimertinib plus selpercatinib led to objective tumor responses in five of six evaluable patients and was well tolerated, with no overlapping toxicities requiring treatment discontinuation (4).

These findings underscore the biological significance and clinical relevance of RET fusions as a mechanism of acquired resistance in EGFR-mutant NSCLC. They provide a strong rationale for the development and implementation of dual inhibition strategies aimed at concurrently targeting both the EGFR and RET signaling pathways, with the potential to overcome resistance, enhance therapeutic efficacy, and improve patient outcomes in this molecularly defined subset of NSCLC.

Here, we present the case of a patient with advanced lung adenocarcinoma harboring an EGFR exon 19 deletion (E746_A750del) who developed a RET fusion-mediated resistance following disease progression on first-line osimertinib. The patient was subsequently treated with off-label osimertinib plus selpercatinib, resulting in meaningful clinical benefit and manageable toxicity. In addition to the case description, we provide a focused review of the literature on RET fusions as an emerging resistance pathway in EGFR-mutant NSCLC and discuss evolving therapeutic strategies to address this mechanism of treatment failure.

## 2 Case report

A 74-year-old Caucasian, never-smoker woman with a medical history of arterial hypertension and diabetes mellitus type 2 was diagnosed in May 2018 as having locally advanced adenocarcinoma with a mass in the left upper lobe measuring <2 cm with mediastinal and ipsilateral supraclavicular lymph nodes [cT1b cN3 cM0-according to the 8th edition of the American Joint Committee on Cancer (AJCC)]. A subsequent ^18^F-fluorodeoxyglucose positron emission tomography/computed tomography (^18^F-FDG PET/CT) scan confirmed additional hypermetabolic activity of the lung lesion as well as to the mediastinal and ipsilateral supraclavicular lymph nodes. Magnetic resonance imaging (MRI) of the brain did not reveal brain metastases. At this time, no distant site of metastasis was identified.

Based on the clinical staging of the disease (cT1bN3M0, Stage IIIB, according to the 8th edition of the AJCC staging system), tumor characteristics, and established treatment guidelines, the multidisciplinary team (MDT) recommended concomitant chemoradiotherapy (cCRT) with cisplatin and pemetrexed as the standard-of-care approach for our patient with unresectable, locally advanced NSCLC.

After approximately 2 years of concomitant chemoradiotherapy, the disease progressed in the lungs. At that time, a re-biopsy of the lung lesion and next-generation sequencing (NGS) (AmpliSeq Comprehensive Panels v3, Illumina) showed an EGFR exon 19 deletion. In the absence of feasible local treatment options, the MDT recommended systemic therapy with osimertinib based on the detection of a sensitizing EGFR exon 19 deletion, and the patient initiated osimertinib 80 mg once daily in October 2020. Osimertinib was well tolerated, with a low incidence of drug-related adverse events such as diarrhea grade 1, dry skin grade 1, and dermatitis acneiform. The patient has thus far remained on osimertinib for >2 years without radiographic evidence of progression.

In December 2023, ^18^F-FDG PET/CT revealed progression in the lung, lymph nodes, and pleural effusion. At that time, NGS performed on the malignant pleural effusion revealed the presence of the EGFR Ex19del, an acquired NCOA4 *–RET* fusion, and a co-mutation in tumor protein p53 (TP53) gene.

**Figure 1:**
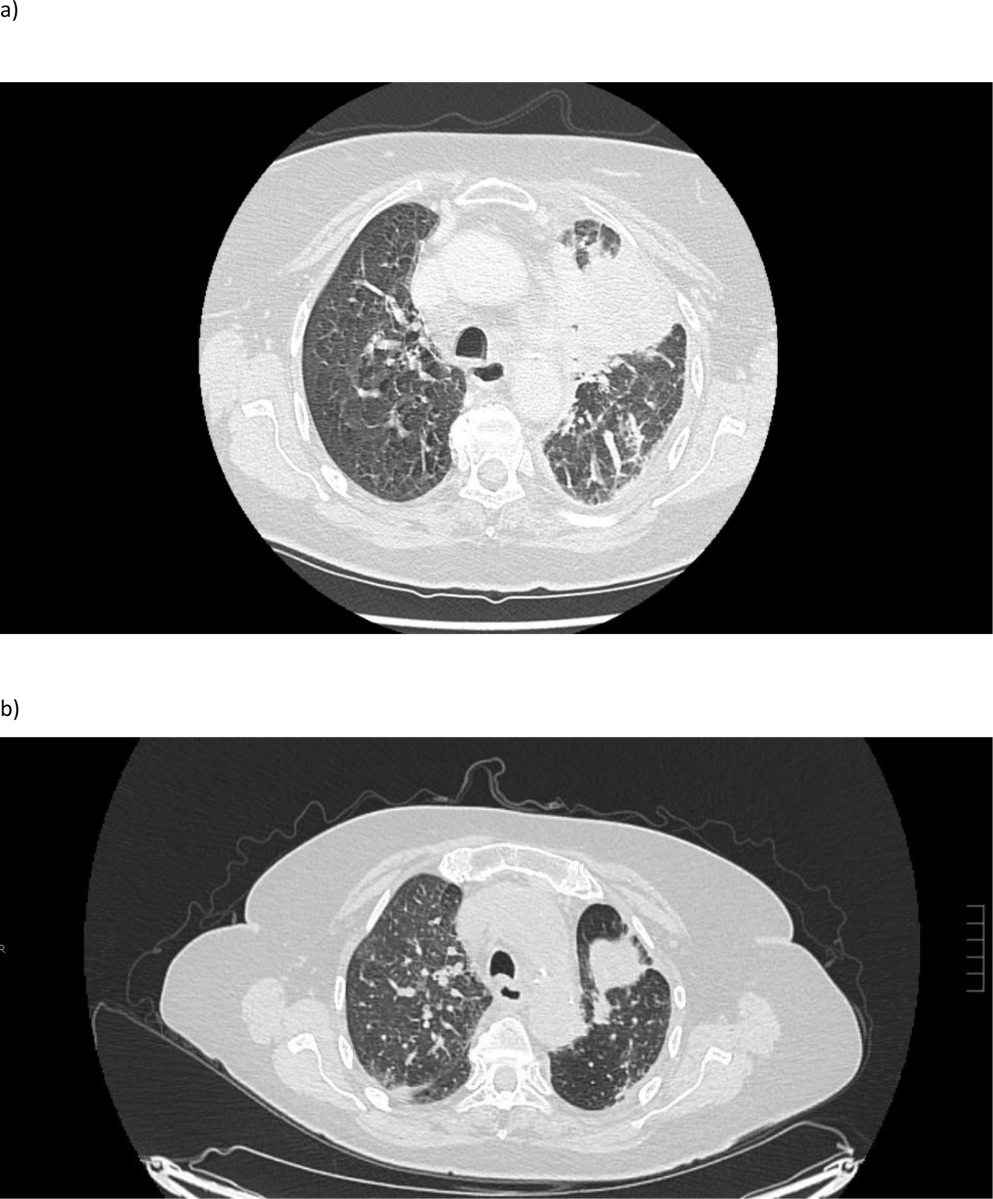
^18^F-FDG PET/CT scan at diagnosis before **(a)** and after treatment with osimertinib in combination with selpercatinib **(b)**.

In January 2024, selpercatinib (80 mg BD) was added to osimertinib (80 mg QD) following disease progression detected by ^18^F-FDG PET/CT in December 2023 and the detection of an acquired NCOA4–RET fusion, along with a TP53 co-mutation in the NGS analysis of the malignant pleural effusion. Given preclinical and clinical evidence supporting the efficacy of dual EGFR and RET inhibition, combination therapy with osimertinib and selpercatinib was initiated to overcome RET-driven resistance while maintaining EGFR-targeted treatment (4). The first assessment performed after 8 weeks revealed a marked response (**Figure 1**). The combination was well tolerated with only grade 1 toxicities including fatigue, hypertension, xerostomia, increased creatinine, and dermatitis acneiform, consistent with the side effect profiles of both drugs as monotherapy. There were no treatment-related serious adverse events.

**Figure 2:**
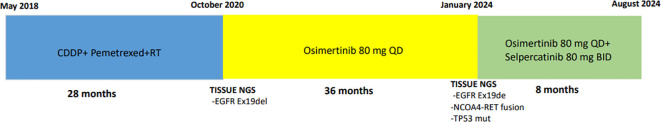
Timeline of the whole course of treatment. NGS, next-generation sequencing.

In August 2024, the patient died due to an unrelated pneumonia. **Figure 2** summarizes the whole course of treatment.

Despite the eventual fatal outcome due to an unrelated pneumonia, this case highlights the importance of personalized medicine in NSCLC, demonstrating that rationally designed combination therapies can extend treatment efficacy, improve symptom management, and maintain patient quality of life, even in the setting of acquired resistance.

## 3 Discussion

This case illustrates the emergence of an *NCOA4*–*RET* fusion as an acquired resistance mechanism in a patient with *EGFR* exon 19 deletion-positive NSCLC following first-line treatment with osimertinib. The clinical benefit observed with the combination of osimertinib and selpercatinib highlights the therapeutic potential of targeting acquired gene fusions in EGFR-mutant NSCLC and underscores the growing importance of repeat molecular profiling at the time of disease progression.

Third-generation EGFR-TKIs such as osimertinib have transformed the management of EGFR-mutant NSCLC. Osimertinib selectively inhibits both sensitizing EGFR mutations (exon 19 deletions and L858R) and the resistance-associated T790M mutation, and it demonstrates enhanced central nervous system (CNS) penetration (5). The FLAURA trial established osimertinib as the preferred first-line agent, with a median progression-free survival (PFS) of 18.9 months compared to 10.2 months with earlier-generation TKIs (2). However, most patients eventually develop resistance, with progression typically occurring approximately 20–22 months (3).

The molecular mechanisms driving resistance to osimertinib are diverse and may involve EGFR-dependent (e.g., C797S, L718Q, or G724S mutations) or EGFR-independent mechanisms and bypass track mechanisms, such as MET amplification, HER2 amplification, PIK3CA mutations, BRAF mutations, KRAS mutations, and histologic transformation to small-cell lung cancer (SCLC) (6–8). Among these, oncogenic fusions—including RET, anaplastic lymphoma kinase (ALK), ROS proto-oncogene 1, receptor tyrosine kinase (ROS1), fibroblast growth factor receptor 3 (FGFR3), and neurotrophic receptor tyrosine kinase (NTRK)—represent an emerging class of targetable alterations that may drive acquired resistance, albeit at a relatively low frequency (<1%–2%) (9, 10).

RET fusions lead to constitutive activation of the RET tyrosine kinase domain, promoting cell proliferation, survival, and resistance to upstream pathway blockade. In the context of EGFR-mutant NSCLC, RET fusions have been shown to activate downstream signaling via mitogen-activated protein kinase (MAPK) and phosphoinositide 3-kinase (PI3K) pathways, bypassing EGFR inhibition and contributing to therapeutic failure (11, 12). The *NCOA4*–*RET* fusion observed in our patient involves the fusion of the coiled-coil domain of *NCOA4* with the tyrosine kinase domain of *RET*, resulting in constitutive dimerization and kinase activation—a mechanism analogous to the more common *KIF5B–RET* and *CCDC6–RET* fusions (13).

Rotow et al. were the first to systematically describe RET fusions as acquired resistance events in EGFR-mutant NSCLC treated with osimertinib. In their multicenter cohort, 14 patients developed RET fusions following progression, with *KIF5B–RET*, *CCDC6–RET*, and *NCOA4*–*RET* as the most prevalent partners (4). Importantly, a subset of these patients received the combination of osimertinib and the selective RET inhibitor selpercatinib, with encouraging clinical responses and manageable toxicity, providing a proof-of-concept for dual EGFR–RET inhibition in this setting.

Since then, several case reports and small series have supported this therapeutic approach. For example, Offin et al. described a patient with an acquired *KIF5B–RET* fusion who responded to the osimertinib–selpercatinib combination with disease control and tolerable side effects (14). Lo Russo et al. similarly reported durable disease stabilization in a patient with a *CCDC6–RET* fusion treated with osimertinib and pralsetinib (15). In preclinical models, combination therapy has been shown to suppress both EGFR- and RET-driven signaling, resulting in enhanced tumor regression compared to monotherapy (16).

Our case is consistent with these findings, demonstrating a rapid and sustained clinical improvement upon the addition of selpercatinib to ongoing osimertinib therapy. The radiologic response was accompanied by symptomatic relief, and the safety profile was favorable, with only grade 1 treatment-emergent adverse events—consistent with prior data on selpercatinib monotherapy (17). This supports the notion that combination targeted therapy can be effective and tolerable, even outside of clinical trial settings.

RET fusions in EGFR-mutant NSCLC are rare, and their detection requires sensitive and comprehensive genomic profiling. Deoxyribonucleic acid (DNA)-based NGS panels may miss certain fusion events due to limitations in intronic coverage; therefore, ribonucleic acid (RNA)-based NGS or hybrid DNA/RNA panels are increasingly recommended for fusion detection (18). In our case, the *NCOA4–RET* fusion was identified from malignant pleural effusion using a hybrid-capture NGS assay, highlighting the utility of liquid- or fluid-based sampling when tissue is not available.

Although no prospective trials have yet defined a standard treatment approach for RET-mediated resistance in EGFR-mutant NSCLC, ongoing studies aim to address this gap. The *ORCHARD* trial (NCT03944772) is a prospective, biomarker-directed platform study evaluating various combination strategies in patients with progressive disease on first-line osimertinib (19). One cohort includes patients with RET fusions treated with osimertinib plus selpercatinib. Additionally, the *LIBRETTO-431* (NCT04194944) and *ARROW* (NCT03037385) trials, while primarily focused on treatment-naïve RET fusion-positive NSCLC, may yield insights relevant to the acquired resistance setting (20, 21).

Recent studies have further elucidated the role of RET fusions as a mechanism of acquired resistance to osimertinib in EGFR-mutant NSCLC. In a *post-hoc* analysis of the AURA3 trial, Chmielecki et al. identified RET fusions among a spectrum of resistance mechanisms, alongside more common alterations such as MET amplification and EGFR C797X mutations, highlighting the molecular heterogeneity underlying treatment failure (22). In their comprehensive review, Passaro and colleagues discuss the evolving landscape of resistance mechanisms to EGFR-TKIs in EGFR-mutant NSCLC and strategies to overcome them. The authors categorize resistance into EGFR-dependent mechanisms (e.g., secondary mutations like T790M and C797S), bypass pathway activation (e.g., MET or HER2 amplification, KRAS mutations, and BRAF mutations), and histologic transformation (e.g., small-cell lung cancer). The review emphasizes the dynamic and often polyclonal nature of resistance, highlighting the need for repeated molecular profiling to guide personalized treatment strategies. It also explores current and emerging therapeutic approaches, including combination regimens, next-generation EGFR inhibitors, and antibody–drug conjugates (ADCs). Importantly, the authors advocate for biomarker-driven clinical trials to optimize sequencing and combination strategies in overcoming resistance (23). These findings underscore the importance of repeat molecular profiling at disease progression and support the integration of combination targeted therapies to overcome on-target and bypass resistance mechanisms in EGFR-mutant NSCLC (24).

A critical consideration moving forward is the potential for resistance to dual EGFR–RET blockade. Emerging data suggest that resistance mutations in RET—such as solvent front mutations (e.g., G810R/S/C)—may limit the efficacy of selpercatinib or pralsetinib over time (25). In such scenarios, second-generation RET inhibitors (e.g., TPX-0046 and LOXO-260) or combinations with downstream pathway inhibitors may be required.

## 4 Conclusion

This case highlights the clinical relevance of RET fusions as a mechanism of acquired resistance to osimertinib in EGFR-mutant NSCLC and supports the use of combination therapy with selpercatinib and osimertinib as a rational and tolerable treatment strategy. As genomic profiling becomes increasingly integrated into routine clinical care, identifying rare but actionable alterations such as RET fusions will be essential for delivering personalized therapy. Future clinical trials and real-world studies are needed to refine therapeutic algorithms, assess the durability of response, and define resistance pathways to EGFR–RET co-inhibition.

## Data Availability

The datasets presented in this study can be found in online repositories. The names of the repository/repositories and accession number(s) can be found in the article/Supplementary Material.
